# Examination of multiple *UGT1A* and *DPYD* polymorphisms has limited ability to predict the toxicity and efficacy of metastatic colorectal cancer treated with irinotecan-based chemotherapy: a retrospective analysis

**DOI:** 10.1186/s12885-017-3406-2

**Published:** 2017-06-20

**Authors:** Dan Liu, Jian Li, Jing Gao, Yanyan Li, Rui Yang, Lin Shen

**Affiliations:** Key Laboratory of Carcinogenesis and Translational Research (Ministry of Education), Department of Gastrointestinal Oncology, Peking University Cancer Hospital & Institute, No. 52, Fucheng Road, Haidian District, Beijing, 100142 China

**Keywords:** Irinotecan, *UGT1A* polymorphisms, *DPYD* polymorphisms, Metastatic colorectal cancer, Toxicity, Clinical response

## Abstract

**Background:**

To evaluate a new *UGT1A* and *DPYD* polymorphism panel to better predict irinotecan-induced toxicity and the clinical response in Chinese patients with metastatic colorectal cancer (mCRC).

**Methods:**

The genotypes of *UGT1A* (UGT1A1*6, UGT1A1*27, UGT1A1*28, UGT1A7*2, UGT1A7*3, UGT1A7*4 and UGT1A9*22) and DPYD (DPYD*5, DPYD c.1896 T > C, and DPYD*2A) were examined by direct sequencing in 661 mCRC patients receiving irinotecan-based chemotherapy. The influences of *UGT1A* and *DPYD* polymorphisms on severe irinotecan-induced toxicities and clinical outcomes were assessed.

**Results:**

In the cohort studied here, the incidence of UGT1A1*6, UGT1A1*28, UGT1A7*2, UGT1A7*3, UGT1A9*22, DPYD*5, and *DPYD c.1896 T > C* variants were 34.8%, 24.2%, 34.3%, 39.4%, 81.8%, 48.4% and 20.4%, respectively. UGT1A1*27 and DPYD*2A had low frequencies and UGT1A7*4 was not found. A total of 59 patients (8.9%) suffered severe diarrhea and 136 patients (20.6%) suffered severe neutropenia. UGT1A1*28 heterozygotes (OR = 2.263, 95%CI 1.395–3.670), UGT1A1*28 homozygotes (OR = 5.910, 95%CI 1.138–30.672) and UGT1A1*6 homozygotes (OR = 4.737, 95%CI 1.946–11.533) were independent risk factors for severe neutropenia. *UGT1A* polymorphisms were not found to relate to severe diarrhea. DPYD*5 was determined to be an independent risk factor for severe diarrhea (OR = 2.143, 95%CI 1.136–4.041). Neither DPYD*5 nor *DPYD c.1896 T > C* was found to relate to severe neutropenia. In the first-line irinotecan-based treatment, UGT1A1*28 and DPYD*5 contributed to higher response rates (*P* = 0.043 and *P* = 0.019, respectively), while DPYD*5 was found to associate with better progression-free survival (*P* = 0.015). UGT1A1*27 contributed to worse overall survival (*P* < 0.001).

**Conclusion:**

Results still showed UGT1A1*6 and UGT1A1*28 to be partially associated with irinotecan-induced toxicity and clinical response. An examination of more *UGT1A* loci, except for UGT1A1*6 and UGT1A1*28, was not helpful to improve the predictive value of irinotecan-based toxicity and efficacy. An examination of DPYD*5 assisted in the prediction of severe diarrhea.

**Electronic supplementary material:**

The online version of this article (doi:10.1186/s12885-017-3406-2) contains supplementary material, which is available to authorized users.

## Background

Irinotecan is currently one of most important drugs in the management of metastatic colorectal cancer (mCRC) [1, 2]. Although the response rate and overall survival are greatly improved with the drug, about 30–50% of patients suffer severe toxicity, which particularly causes neutropenia and diarrhea [2]. *UGT1A* polymorphisms, especially UGT1A1*6 and UGT1A1*28, were previously noted to predict irinotecan-induced toxicity, but the results were inconstant [3, 4]. Based on a previous study performed in our center [5], UGT1A1*6 and UGT1A1*28 were found to be related solely to irinotecan-induced severe neutropenia, and not to diarrhea, as most studies in Asia indicated [4, 6]. The predictive sensitivity and specificity were relatively low, only 37.6% and 61.6%, respectively. Although the combined examination of multiple *UGT1A* loci improved the predictive sensitivity and specificity to irinotecan-induced toxicity, it still focused on predictability for severe neutropenia [7]. The results were based on studies with small samples. In actual clinical practice, severe diarrhea was more closely associated with mortality than neutropenia [8], but there is still no definite biomarker that can predict severe diarrhea in Asian patients [9, 10]. Moreover, irinotecan is commonly used in combination with fluorouracil, which also induces severe neutropenia and diarrhea. *DPYD* polymorphisms, which are associated with fluorouracil levels in vivo, are associated with the occurrence of fluorouracil-induced toxicity [11, 12]. In this way, it is necessary to find ways to improve the predictability of combined *UGT1A* with an examination of *DPYD* polymorphisms. This is the first large sample analysis of a combined examination of both *UGT1A* and *DPYD* polymorphisms to predict irinotecan-based chemotherapy-induced toxicity and the clinical response in Chinese patients.

This study was designed to evaluate the combinations of *UGT1A* and *DPYD* polymorphisms in predicting the occurrence of treatment-induced toxicity, clinical response and survival in China. Because of regional ethnic diversity, the genotype distribution differs in various parts of China. Based on the genotype frequency distribution in Chinese and other Asian patients from previous studies [13–16], the genotypes of 661 patients have been examined at 9loci: UGT1A1*6, UGT1A1*27 (c.686C > A), UGT1A1*28, UGT1A7*2 (c.387 T > G), UGT1A7*3 (c.387 T > G, c.622 T > C), UGT1A7*4 (c.622 T > C), UGT1A9*22 (−118 T9 > T10), DPYD*5 (c.1627A > G), DPYD*2A (c.1905 + 1G > A), and *DPYD c.1896 T > C.* The relationship of each genotype to the risk of treatment-induced toxicities, response rate and overall survival are explored here. These findings may be used to establish a new panel, that would be more efficient in predicting treatment-induced toxicity or efficacy in China.

## Methods

### Patients

**Fig. 1:**
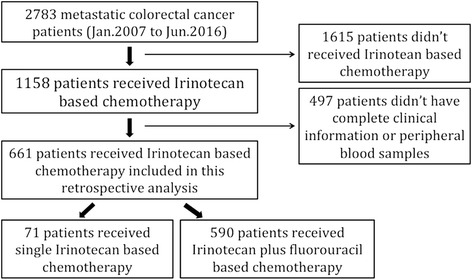
Screening process for analyzed patients. Of 2783 colorectal cancer patients available for screening, 1615 patients who did not received irinotecan-based chemotherapy and 497 patients without complete clinical information and blood samples were excluded. Of the 661 patients included in this analysis, 71 patients received irinotecan plus fluorouracil-based chemotherapy, while the other 590 patients received irinotecan plus fluorouracil-based chemotherapy

A total of 2783 colorectal cancer patients who received chemotherapy at Peking University Cancer Hospital between January 2007 and June 2016 were screened for this retrospective study. Patients eligible for the study met the following criteria: histologically confirmed adenocarcinoma of the colorectum, stage IV disease, they received at least 2 cycles of irinotecan-based chemotherapy unless intolerable toxicity or disease progression occurred, they had peripheral blood samples taken, and complete clinical information was available for toxicity and efficacy evaluation. Patients were excluded from the study based on the following criteria: they received irinotecan-based chemotherapy for adjuvant treatment, and they did not have toxicity and efficacy information available for evaluation. The screening process is shown in Fig. 1.

All patients provided written informed consent for their peripheral blood to be used in this research. This study was approved by the Medical Ethics Committee of Peking University Cancer Hospital and was performed according to the principles of the Declaration of Helsinki.

### Treatment and drug administration

Before patients received the irinotecan-based chemotherapy, routine blood tests of hepatic and renal function and performance status evaluation of each patient were performed and considered to be essential. The regimens in this study included irinotecanalone or combined with target treatment (*n* = 71, irinotecan dosage, 180 mg/m^2^), irinotecan combined with fluorouracil (5-Fu, Capecitabine, S-1 or tegafur) or plus target treatment (*n* = 554, irinotecan dosage, 180 mg/m^2^) and FOLFOXIRI (*n* = 36, irinotecan dosage, 150 mg/m^2^). Each patient received at least 2 cycles of irinotecan-based chemotherapy, unless the patients suffered disease progression or intolerable toxicity. Routine blood tests and an evaluation of adverse events were performed after each administration of irinotecan or before the initiation of the next chemotherapy.

### Toxicity and response assessment

Toxicity was evaluated based on the medical records according to the National Cancer Institute Common Toxicity Criteria for Adverse Events, Version 4.0 (NCI-CTC 4.0 criteria, http://ctep.cancer.gov/reporting/ctc.html; accessed in October 2015). Grade 3 or 4 neutropenia and diarrhea were defined as severe toxicity.

The response rate was evaluated every 2–3 cycles or whenever the patient’s condition changed by imaging evaluation (CT or MRI) according to the response evaluation criteria in solid tumors (RECIST) [17]. All of the survival data were obtained from medical records and telephone follow-up. The last follow-up of recurrence and survival information was August 1, 2016. Progression-free survival (PFS) was identified as the time from the start of chemotherapy to disease progression, the last follow-up, or death of any cause. Overall survival (OS) was defined as the time from the start of irinotecan-based chemotherapy to death.

### Genomic DNAs extraction and genotyping of UGT1A and DPYD

Two-milliliter peripheral blood samples were acquired from metastasis colorectal cancer patients before receiving treatment and stored at −80 °C. The genomic DNA samples were extracted from these blood samples using QLAamp Blood Kit (Qiagen, Hilden, Germany). The fragments of UGT1A (UGT1A1*6, UGT1A1*27, UGT1A1*28, UGT1A7*2, UGT1A7*3, UGT1A7*4 and UGT1A9*22) and DPYD (DPYD*5, DPYD c.1896 T > C and DPYD*2A) were amplified by polymerase chain reaction (PCR). All primers are shown in Table 1. Each 20 ul PCR reaction mixture consisted of 2 ul of 10 × LA PCR buffer II, 2 ul of 10 mmol/L dNTPs, 0.15 ul of LA Taq (DRR200A, Takara), 100–150 ng of genomic DNA, and 0.5 ul of each primer (10umol/L). The PCR conditions of UGT1A1*27 and DPYD*5 were 95 °C for 5 min, 45 cycles of 95 °C for 10 s, 56 °C for 45 s and 72 °C for 20s, and a final extension at 72 °C for 10 min, and a final 4 °C for 10 min. The PCR products were indentified by 2% agarose gel electrophoresis and sequenced using an Invitrogen 3730XL genetic analyzer. The sequencing results were analyzed using Chromas software.

**Table 1:** The primers of UGT1A/DPYD variants genotypes

Mutation Type	PCR Primer (5′-3′)	Product length
UGT1A1*6 [5]	Primer-F: ACGCCTCG TTGTACATCAGAG	217 bp
	Primer-R: CCTTGTT GTGCAGTAAGTGG	
UGT1A1*27	Primer-F: ACTTACTGCACAACAAGGAGCT	484 bp
	Primer-R: CACACCTGGGATAGTGGATTTTG	
UGT1A1*28 [5]	Primer-F: AGCCAGTTCAACTGTTGTTGC	208 bp
	Primer-R: TTTGCT CCTGCCAGAGGTTC	
UGT1A7*2/*3/*4 [28]	Primer-F: TTTGCCGATGCTCGCTGGACG	415 bp
	Primer-R: GCTATTTCTAAGACATTTTTGAAAAAATAGGG	
UGT1A9*22 [35]	Primer-F: ACTTAACATTGCAGCACAGG	556 bp
	Primer-R: ATGGGCAAAAGCCTTGAACT	
DPYD*5	Primer-F: ATTCAGTTCACTGCTCACTGAC	370 bp
	Primer-R: GAGAAAGTTTTGGTGAGGGCA	
*DPYD c.1896 T > C,*	Primer-F: TGGACAAAGCTCCTTTCTGAATA	231 bp
DPYD*2A [36]	Primer-R: CAGCAAAGCAACTGGCAGAT	

### Statistical analysis

Differences between UGT1A and DPYD variants and severe irinotecan-induced toxicity were analyzed using the chi-square and Fisher’s exact tests. The association of genotypes with risk of severe irinotecan induced adverse events was assessed using logistic models. The Back-wald method of multivariate analysis model was used to avoid possible interactions. Survival curves were analyzed using the Kaplan-Meier method and compared by the log-rank test. All analyses were carried out using SPSS version 22.0 (SPSS Inc., Chicago, IL, US). The predictive powers of genotypes were recorded using Odds Ratios(ORs) and 95% confidence internals (CIs). All statistical analyses were two-sided testsand *P* values <0.05 were considered to be statistically significant.

## Results

There were 661 mCRC patients who were finally enrolled in this study (all of the clinical data and the patients’ genotypes of *UGT1A* and *DPYD* are shown in the Additional file 1). Of the study population, 406 patients (61.4%) were male and 255 patients (38.6%) were female, and the median age was 56 years old (interquartile range [IQR] 47, 63). There were 98 patients (14.8%) who received irinotecan-based regimens as the first-line treatment and 563 patients (85.2%) who received irinotecan-based regimens as the second-line treatment or further. There were 71 patients (10.7%) who received single irinotecan-based chemotherapy and 590 patients (89.3%) who received irinotecan plus fluorouracil-based chemotherapy. All patients were eligible for toxicity evaluation and 634 patients were eligible for response evaluation. The incidence of severe diarrhea and neutropenia was 8.9% (*n* = 59) and 20.6% (*n* = 136), respectively. During the follow-up, 512 patients had disease progression and 346 patients were dead.

Among all of the patients, 49 of 71 patients who received single irinotecan-based chemotherapy had all of the *UGT1A* polymorphism loci examined, while 496 of 590 patients who received irinotecan plus fluorouracil-based chemotherapy had all of the *UGT1A* and *DPYD* polymorphism loci examined. The remaining 116 patients (including 22 patients who received irinotecan plus fluorouracil-based chemotherapy) only finished an examination of UGT1A1*6 and UGT1A1*28, because of examination failure and sample depletion. The genotypes are shown in Table 2.

**Table 2:** Genotypes of *UGT1A* and *DPYD* in mCRC patients

Genotypes	No. of patients	%
UGT1A1*6		
G/G	431	65.2%
G/A	198	30.0%
A/A	32	4.8%
UGT1A1*27		
C/C	535	98.2%
C/A	10	1.8%
UGT1A1*28		
TA6/TA6	501	75.8%
TA6/TA7	152	23.0%
TA7/TA7	8	1.2%
*UGT1A7*		
UGT1A7*1/*1	196	36.0%
UGT1A7*1/*2	103	18.9%
UGT1A7*1/*3	131	24.0%
UGT1A7*2/*2	31	5.7%
UGT1A7*2/*3	53	9.7%
UGT1A7*3/*3	31	5.7%
UGT1A9*22		
T9/T9	99	18.2%
T9/T10	442	81.1%
T10/T10	4	0.7%
DPYD*5		
A/A	256	51.6%
A/G	199	40.1%
G/G	41	8.3%
DPYD*2A		
G/G	495	99.8%
G/A	1	0.2%
*DPYD c.1896 T > C*		
T/T	395	79.6%
T/C	96	19.4%
C/C	5	1.0%

### Analysis of chemotherapy-induced toxicities

In this retrospective study, sex, age, primary tumor location [18], chemotherapy regimens, line of treatment, and *UGT1A* and *DPYD* polymorphisms were included in the analysis (Table 3). Two loci, UGT1A7*4 and DPYD*2A, were excluded, due to their low frequency. The severe neutropenia incidence was 24.7% in females, and 18.0% in males, with *P* value of 0.056 in multivariate analysis. There were 30.6% of patients who suffered severe neutropenia in the first-line treatment, while 18.8% of patients suffering severe neutropenia in the second-line treatment or further, with a *P* value 0.009 in multivariate analysis. DPYD*5 was the independent predictive factor of severe diarrhea (OR = 2.143, 95%CI 1.136–4.041). UGT1A1*28 heterozygotes (OR = 2.263, 95%CI 1.395–3.670), UGT1A1*28 homozygotes (OR = 5.910, 95%CI 1.138–30.682) and UGT1A1*6 homozygotes (OR = 4.737, 95%CI 1.946–11.533) were the independent predictive factors of severe neutropenia.

**Table 3:** Univariate and multivariate analysis of chemotherapy induced toxicity

	Severe diarrhea				Severe neutropenia			
Factors	Univariate analysis		Multivariate analysis		Univariate analysis		Multivariate analysis	
	N/Total(%)	*P* value	OR(95%CI)	*P* value	N/Total(%)	*P* value	OR(95%CI)	*P* value
Sex								
Male	39/406(9.6%)				73/406(18.0%)			
Female	20/255(7.8%)	0.439	NA^a^	NA	63/255(24.7%)	0.037	1.538(0.989–2.393)	0.056
Age								
≦65y	44/545(8.1%)				119/545(21.8%)			
>65y	15/116(12.9%)	0.096	NA	NA	17/116(14.7%)	0.082	NA	NA
Primary tumor location^b^								
Left-side colorectum	48/487(9.9%)				97/487(19.9%)			
Right-side colon	11/174(6.3%)	0.160	NA	NA	39/174(22.4%)	0.485	NA	NA
Chemotherapy regimens								
Single IRI based regimens	8/71(11.3%)				9/71(12.7%)			
IRI^c^ + fluorouracil based regimens	51/590(8.6%)	0.464	NA	NA	127/590(21.5%)	0.081	NA	NA
Line of treatment								
First line treatment	9/98(9.2%)				30/98(30.6%)			
≧Second line treatment	50/563(8.9%)	0.923	NA	NA	106/563(18.8%)	0.008	0.504(0.301–0.845)	0.009
UGT1A1*6								
G/G	42/431(9.7%)				79/431(18.3%)			
G/A	14/198(7.1%)				43/198(21.7%)		1.376(0.854–2.215)	0.189
A/A	3/32(9.4%)	0.548	NA	NA	14/32(43.8%)	0.002	4.737(1.946–11.533)	0.001
UGT1A1*27								
C/A	0/10(0.0%)				109/535(20.4%)			
C/C	53/535(9.9%)	0.609	NA	NA	2/10(20.0%)	0.977	NA	NA
UGT1A1*28								
TA6/TA6	40/501(8.0%)				90/501(18.0%)			
TA6/TA7	18/152(11.8%)				42/152(27.6%)		2.263(1.395–3.670)	0.001
TA7/TA7	1/8(12.5%)	0.323	NA	NA	4/8(50.0%)	0.004	5.910(1.138–30.682)	0.034
*UGT1A7*								
UGT1A7*1/*1,*1/*2,*2/*2	31/330(9.4%)				54/330(16.4%)			
UGT1A7*1/*3,*2/*3,*3/*3	22/215(10.2%)	0.747	NA	NA	57/215(26.5%)	0.004	NA	NA
UGT1A9*22								
T9/T9	10/99(10.1%)				26/99(26.3%)			
T9/T10,T10/T10	43/446(9.6%)	0.889	NA	NA	85/446(19.1%)	0.107	NA	NA
DPYD*5								
A/A	16/256(6.3%)				53/256(20.7%)			
A/G	30/240(12.5%)	0.016	2.143(1.136–4.041)	0.019	51/240(21.3%)	0.881	NA	NA
*DPYD c.1896 T > C*								
T/T	32/395(8.1%)				84/395(21.3%)			
T/T,T/C	14/101(13.9%)	0.075	NA	NA	20/101(19.8%)	0.747	NA	NA

^a^: Non-acquired; ^b^: Left-side colorectum included splenic flexure, descending colon, sigmoid colon and rectum; Right-side colon included cecum, ascending and transverse colon [18]; ^c^: Irinotecan

Out of all of the patients who received irinotecan-based chemotherapy, those who have more mutational alleles of UGT1A1*6 and UGT1A1*28 were found to be more likely to suffered severe toxicity (*P* = 0.001), especially severe neutropenia (*P* < 0.001). The predictive sensitivity and specificity of *UGT1A* polymorphisms were 32.4% and 53.1%, respectively. Of the patients who received irinotecan plus fluorouracil-based chemotherapy, we analyzed the severe toxicity risk based on UGT1A1*6/*28 and DPYD*5 panels. More mutational alleles of UGT1A1*6/*28 and DPYD*5 were also revealed to had increased incidence of severe neutropenia (*P* = 0.008). And patients with ≧3 mutation alleles had higher risk of suffering severe diarrhea, with the incidence of 15.9%, but without significant *P* value. The predictive sensitivity and specificity of UGT1A*6/*28 and DPYD*5 panels were 33.1% and 85.3%, respectively (Table 4).

**Table 4:** Correlation of UGT1A polymorphisms with severe toxicity

	Severe diarrhea		Severe neutropenia		Severe toxicity	
Genotype	N/Total(%)	*P* value	N/Total(%)	*P* value	No.(%)	*P* value
UGT1A1*6/*28 panels (*N* = 661)						
Wild type^a^	31/368(8.4%)		59/368(16.0%)		84/368(22.8%)	
Single allele variants^b^	23/228(10.1%)		53/228(23.2%)		66/228(28.9%)	
≧2 alleles variants^c^	5/65(7.7%)	0.736	24/65(36.9%)	<0.001	29/65(44.6%)	0.001
UGT1A1*6/*28 and DPYD*5 panels (*N* = 496)						
Wild type^d^	5/114(4.4%)		17/114(14.9%)		20/114(17.5%)	
Single allele variants^e^	22/214(10.3%)		36/214(16.8%)		54/214(25.2%)	
2 alleles variants^f^	12/124(9.7%)		37/124(29.8%)		44/124(35.5%)	
≧3 alleles variants^g^	7/44(15.9%)	0.147	14/44(31.8%)	0.008	21/44(47.7%)	0.001

^a^patients with genotype: G/G and TA6/TA6. ^b^patients with genotype: G/A, and TA6/TA6; or G/G and TA6/TA7. ^c^patients with genotype: A/A and TA6/TA6; or G/A and TA6/TA7; or G/G and TA7/TA7; or A/A and TA6/TA7. ^d^patients with genotype: G/G, TA6/TA7 and A/A. ^e^patients with genotype: G/A, TA6/TA6 and A/A; or G/G, TA6/TA7 and A/A; or G/G, TA6/TA6, A/G. ^f^patients with genotype: G/A, TA6/TA7 and A/A; or G/G, TA6/TA6 and G/G; or G/G, TA7/TA7 and A/A; A/A, TA6/TA6 and A/A. ^g^patients with genotype: G/A, TA6/TA7 and A/G; or A/A, TA6/TA6 and A/G; or G/G, TA7/TA7 and G/G; or G/A, TA6/TA7 and G/G; or A/A, TA6/TA6 and G/G

### Analysis of chemotherapy clinical response

The clinical response of irinotecan-based chemotherapy varied across different lines of treatment. In the first-line treatment group of patients, 5 patients were not available to evaluate efficacy due to stopping chemotherapy for intolerable toxicity. Only 4 patients received single irinotecan-based chemotherapy as a first-line treatment, because of old age or bad performance. The objective response rate (ORR) was 32.3% (30/93). For the second-line treatment or further, 22 patients could not evaluate efficacy due to stopping chemotherapy for intolerable toxicity. The objective rate was 12.2% (66/541). Of the patients who received irinotecan plus fluorouracil-based chemotherapy as the first-line treatment, UGT1A1*28 and DPYD*5 contributed to a higher ORR. Neither clinical factors (including sex, age, and primary tumor location) nor *UGT1A/DPYD* polymorphisms were related to the disease control rate (DCR) in any line of treatment (Table 5).

**Table 5:** UGT1A/DPYD polymorphisms and clinical response

Genotype	Single IRI ± targeted therapy								IRI + fluorouracill ± targeted therapy							
	First line treatment				≧Second line treatment				First line treatment				≧Second line treatment			
	ORR	*P* value	DCR	*P* value	ORR	*P* value	DCR	*P* value	ORR	*P* value	DCR	*P* value	ORR	*P* value	DCR	*P* value
Sex																
Male	0/1 (0.0%)		1/1 (100.0%)		0/29 (0.0%)		18/29 (62.1%)		17/56 (30.4%)		49/56 (87.5%)		39/295 (13.2%)		235/295 (79.7%)	
Female	1/3 (33.3%)	1.000	2/3 (67.7%)	1.000	4/34 (11.8%)	0.118	19/34 (55.9%)	0.619	12/33 (36.4%)	0.559	29/33 (87.9%)	0.958	23/183 (12.6%)	0.837	141/183 (77.0%)	0.498
Age																
≦65y	1/3 (33.3%)		2/3 (66.7%)		4/42 (9.5%)		28/42 (66.7%)		28/80 (35.0%)		71/80 (88.8%)		56/398 (14.1%)		317/398 (79.6%)	
>65y	0/1 (0.0%)	1.000	1/1 (100.0%)	1.000	0/21 (0.0%)	0.292	9/21 (42.9%)	0.070	1/9 (11.1%)	0.216	7/9 (77.8%)	0.343	6/80 (7.5%)	0.11	59/80 (73.8%)	0.24
Primary tumor location																
Left-side colorectum	1/3 (33.3%)		2/3 (66.7%)		3/43 (7.0%)		27/43 (62.8%)		20/60 (33.3%)		54/69 (90.0%)		47/362 (13.0%)		289/362 (79.8%)	
Right-side colon	0/1 (0.0%)	1.000	1/1 (100.0%)	1.000	1/20 (5.0%)	1.000	10/20 (50.0%)	0.337	9/29 (31.0%)	0.828	24/29 (82.8%)	0.331	15/116 (12.9%)	0.988	87/116 (75.0%)	0.269
UGT1A1*6																
G/G	1/4 (25.0%)		3/4 (75.0%)		3/46 (6.5%)		28/46 (60.9%)		17/54 (31.5%)		49/54 (90.7%)		42/310 (13.5%)		239/301 (77.1%)	
G/A	0/0 (0.0%)		0/0 (0.0%)		1/15 (6.7%)		7/15 (46.7%)		9/31 (29.0%)		25/31 (80.6%)		16/145 (11.0%)		119/145 (82.1%)	
A/A	0/0 (0.0%)	NA	0/0 (0.0%)	NA	0/2 (0.0%)	0.932	2/2 (100.0%)	0.302	3/4 (75%)	0.175	4/4 (100.0%)	0.295	4/23 (17.4%)	0.615	18/23 (78.3%)	0.483
UGT1A1*27																
C/A	0/0 (0.0%)		0/0 (0.0%)		3/43 (7.0%)		28/43 (65.1%)		2/3 (66.7%)		2/3 (66.7%)		54/387 (14.0%)		313/387 (80.9%)	
C/C	1/2 (50.0%)	NA	2/2 (100.0%)	NA	0/0 (0.0%)	NA	0/0 (0.0%)	NA	26/84 (31.0%)	0.193	74/84 (88.1%)	0.272	0/7 (0.0%)	0.600	6/7 (85.7%)	0.747
UGT1A1*28																
TA6/TA6	1/3 (33.3%)		2/3 (66.7%)		4/48 (8.3%)		29/48 (60.4%)		15/59 (25.4%)		52/59 (88.1%)		53/373 (14.2%)		293/373 (78.6%)	
TA6/TA7,TA7/TA7	0/1 (0.0%)	1.000	1/1 (100.0%)	1.000	0/15 (0.0%)	0.564	8/15 (53.3%)	0.627	14/30 (46.7%)	0.043	26/30 (86.7%)	0.842	9/105 (8.6%)	0.129	83/105 (79.0%)	0.913
*UGT1A7*																
UGT1A7*1/*1,*1/*2,*2/*2	1/2 (50.0%)		2/2 (100.0%)		3/32 (9.4%)		20/32 (62.5%)		12/47 (25.5%)		42/47 (89.4%)		35/241 (14.5%)		194/241 (80.5%)	
UGT1A7*1/*3,*2/*3,*3/*3	0/0 (0.0%)	0.558	0/0 (0.0%)	NA	0/11 (0.0%)	0.558	8/11 (72.7%)	0.539	15/38 (39.5%)	0.17	32/38 (84.2%)	0.482	19/153 (12.4%)	0.554	125/153 (81.7%)	0.767
UGT1A9*22																
T9/T9	0/0 (0.0%)		0/0 (0.0%)		0/8 (0.0%)		5/8 (62.5%)		6/13 (46.2%)		11/13 (84.6%)		5/71 (7.0%)		61/71 (85.9%)	
T9/T10,T10/T10	1/2 (50.0%)	1.000	2/2 (100.0%)	NA	3/35 (8.6%)	1.000	23/35 (65.7%)	0.863	21/72 (29.2%)	0.226	63/72 (87.5%)	0.776	49/323 (15.2%)	0.071	258/323 (79.9%)	0.241
DPYD*5																
A/A	NA		NA		NA		NA		8/41 (19.5%)		34/41 (82.9%)		32/211 (15.2%)		174/211 (82.5%)	
A/G,G/G	NA	NA	NA	NA	NA	NA	NA	NA	19/44 (43.2%)	0.019	40/44 (90.9%)	0.273	22/183 (12.0%)	0.365	145/183 (79.2%)	0.415
*DPYD c.1896 T > C*																
T/T	NA		NA		NA		NA		24/69 (34.8%)		60/69 (87.0%)		42/314 (13.4%)		254/314 (80.9%)	
T/T,T/C	NA	NA	NA	NA	NA	NA	NA	NA	3/16 (18.8%)	0.215	14/16 (87.5%)	0.953	12/80 (15.0%)	0.706	65/80 (81.3%)	0.942

IRI: Irinotecan; ORR: Objective response rate; DCR: Disease control rate; Tumor location: Left-side colorectum included splenic flexure, descending colon, sigmoid colon and rectum; Right-side colon included cecum, ascending and transverse colon [18]; NA: Non-acquired

### Analysis of irinotecan-induced progression- free survival and overall survival

**Fig. 2:**
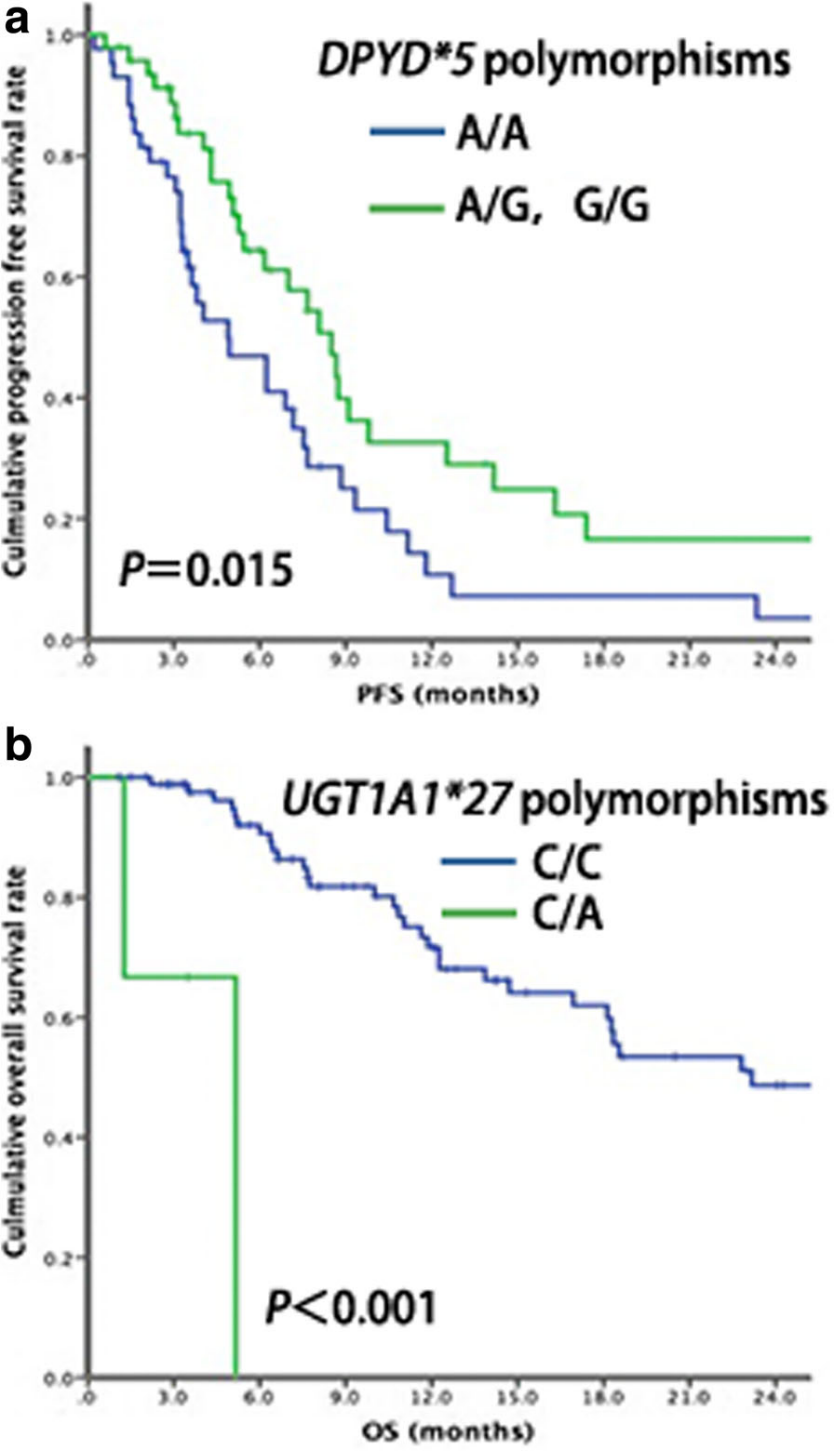
Significant survival curves of PFS and OS. **a**. The survival curves of PFS in different DPYD*5 genotypes; **b** The survival curves of OS in different UGT1A1*27 genotypes

Of the patients who received the first-line irinotecan-based chemotherapy, the median PFS was 7.00 months (IQR 3.30, 11.80). DPYD*5 mutation contributed to better PFS than wild type (4.90 months vs. 8.50 months, *P* = 0.015, Fig. 2a). Patients with the UGT1A1*27 mutation showed a shorter OS than the wild-type patients (5.17 vs. 23.17, *P* < 0.001, Fig. 2b). In the second-line treatment or further, the median PFS was 5.57 months (IQR 2.63, 11.23). Neither UGT1A nor DPYD polymorphisms showed any significant relationship with PFS or OS (all *P* values >0.05).

## Discussion

In this cohort, the incidence of severe diarrhea and neutropenia was 8.9% and 20.8%. These were consistent with the previously reported results at the same center [5]. Clinical factors (including sex, age, primary tumor location, and chemotherapy regimens) did not show a significant relationship with treatment-induced severe diarrhea. Patients who received irinotecan-based chemotherapy as a second-line treatment or further had a lower risk of suffering severe neutropenia. The results are also shown in a previous report [19], which might be explained by more patients with better treatment tolerance receiving the second-line treatment or further. Female patients showed a potentially higher incidence of severe neutropenia, but with no statistical significance; however, in the report of Tsunedomi R et al., being female was an independent risk factor of severe neutropenia [7].

*UGT1A* genotype frequency and the effect on treatment-induced toxicity varied across ethnic groups. Early in 2005, UGT1A1*28 was recognized as a risk factor for irinotecan induced toxicities by the U.S Food and Drug Administration (FDA). In Asia, however, UGT1A1*28 were not applicable to the prediction of irinotecan-induced toxicity because of its low frequency. In this study, the genotype frequency of UGT1A1*6 and UGT1A1*28 were similar to previous reports in Asia [5, 20]. Both UGT1A1*6 and UGT1A1*28 related to G3–4 neutropenia, rather than delayed diarrhea, which was consistent with several large-sample analysis in Asia [4–7]. Several small-sample analyses also noted that UGT1A1*28 and UGT1A1*6 could predict severe irinotecan-induced severe diarrhea [21, 22], which did not appear in the current study. A small sample analysis of Atasilp C et al., involving UGT1A1*6 and UGT1A1*28 were included in this analysis. Although individual UGT1A1*6 and UGT1A1*28 did not show a relationship with severe diarrhea neutropenia, the correlation of UGT1A1*6 and UGT1A1*28 revealed a significant association with severe neutropenia. Correlation of UGT1A1*6 and UGT1A1*28 also showed the same results in this study. In Thai patients, the UGT1A1*28 mutation frequency was nearly the same with Chinese patients (22.8% vs. 24.2%), while the UGT1A1*6 mutation frequency was lower than in Chinese patients (15.9% vs. 34.8%) [23]. The difference of polymorphism frequencies induced by ethnicity might explain the differences in the results of the two studies. UGT1A1*27 is a genotype only in Asians with lower UGT enzyme activity and low frequency [24]. Ten patients (1.8%) had UGT1A1*27 heterozygotes in this cohort, but only 2 patients suffered severe neutropenia. The incidence of severe toxicity was much lower than in previous reports [25]. The genotype frequency of UGT1A9*22 was 81.8% in this study, which was similar to findings reported in Japan. However, the UGT1A9*22 homozygotes in China were much rarer than in Japan (0.7% vs. 34.7%) [26]. UGT1A9*22 did not show an association with irinotecan-induced toxicity in this study. It has previously been reported that UGT1A9*22 variants have a lower risk of suffering irinotecan-induced severe neutropenia [7, 25, 26]. Chinese patients had similar UGT1A7*2/*3 frequency to Japanese patients, but a lower frequency than Greeks [26, 27]. Several studies have shown that UGT1A7*3 is associated with higher risk of suffering severe neutropenia [26–29]. Tziotou M’s study also showed that UGT1A7*3 to be related to severe diarrhea [27]. In this study, UGT1A7*3 had a significant ability to predict severe neutropenia in univariate analysis, but the relationship did not appear to be significant in multivariate analysis. UGT1A7*3 was not an independent biomarker in the prediction of irinotecan-induced toxicity for Chinese patients. Among the patients who received targeted drugs, only UGT1A7*3 was found to be associated with a higher risk of G3–4 neutropenia incidence. Targeted drug treatment might affect the predictability of the toxicity of *UGT1A* polymorphisms. Regimens with different targeted drugs might also affect evaluation of toxicity. The influence of targeted drugs on the relationship between *UGT1A* polymorphisms and toxicity should be further studied. Finally, the results showed that UGT1A1*6 and UGT1A1*28 had an association with irinotecan-induced severe neutropenia. Patients with more mutant variants had a higher risk of suffering severe neutropenia; however, no other significant loci of *UGT1A* polymorphisms were found to set up a new panel to better indicate irinotecan-induced toxicity.

Fluorouracil is generally combined with irinotecan. *DPYD* polymorphisms influenced the activity of dihydropyrimidine dehydrogenase (DPD) considerably, which was associated with fluorouracil’s metabolism and ethnic variation also appeared in *DPYD* polymorphisms [14, 16, 30]. In western countries, it has been reported that DPYD*2A mutant variants contribute to a higher risk of severe toxicity [30]; however, DPYD*2A is rarely found. This was consistent with the findings of this study. Only 1 DPYD*2A heterozygote (0.2%) was found in this analysis. The ability of DPYD*2A to indicate fluorouracil-induced toxicity in China could not be assessed. DPYD*5 and *DPYD c.1896 T > C* had allele frequencies of 28.4% and 10.7%, respectively, which was consistent with previous reports [31]. In this cohort, it was noted that DPYD*5 associated with higher risk of severe diarrhea. However, in Zhang XP et al. and Yamauchi et al.’s study, DPYD*5 related to the incidence of severe neutropenia [31, 32]. In addition, the study of Felicia FS et al. showed that *DPYD c.1896 T > C* independently predicted severe fluorouracil-induced toxicity, which did not happen in this analysis [16]. A combined examination of UGT1A1*6, UGT1A1*28 and DPYD*5 was found to improve the predictive specificity for toxicity compared with an examination of UGT1A1*6 and UGT1A1*28 (53.1% vs. 85.6%) among patients receiving irinotecan plus fluorouracil-based chemotherapy.

The association between *UGT1A* and *DPYD* polymorphisms and clinical outcomes were analyzed, as well as the toxicity. The response rate and survival varied across different treatment lines. Among patients who received irinotecan-based chemotherapy as a first-line treatment, this analysis first noted that UGT1A1*27 contributed to worse OS than wild type variants, although the number of analyzed samples was small. Moreover, UGT1A1*28 contributed to a higher objective response rate, which was consistent with studies reported by Fujita and Toffoli G’s team [25, 33]. While, in Lu CY and colleagues’ study, UGT1A1*28 led to bad clinical outcomes [34]. This might be explained by a large number of factors affecting the survival. Single *UGT1A* gene polymorphisms were found to have only a limited ability to predict survival, and multiple chemotherapy regimens might also be involved. Unlike *UGT1A*, there have only been limited studies assessing the relationship between *DPYD* polymorphisms and survival. In this analysis, DPYD*5 mutant variants predicted better PFS in the first-line treatment of irinotecan plus fluorouracil-based regimens, and *DPYD* polymorphisms were not found to associate with overall survival.

Because this study was a retrospective analysis, bias was unavoidable. The value of this study relies on the large samples’ combined examination for *UGT1A* and *DPYD* polymorphisms. Although it was not possible to establish a new panel to improve the predictability of toxicity in this study, the analysis showed that more attention should be paid to homozygote of UGT1A1*6in the context of irinotecan-induced severe neutropenia, such as constantly monitoring the levels of neutrophile granulocytes and preventive treatment for neutropenia. For this reason, further studies should focus on polymorphisms of other genes related to irinotecan metabolism.

## Conclusion

In brief, only UGT1A1*6 and UGT1A1*28 variants were associated with irinotecan-induced neutropenia, but not with diarrhea. A combined examination of UGT1A1*6, UGT1A1*28 and DPYD*5 were found to improve the predictive specificity of toxicity. *UGT1A* and *DPYD* polymorphisms were still limited to the prediction of clinical response. A combined examination of more *UGT1A* polymorphisms will not be helpful in improving predictive value of irinotecan-induced toxicity.

## Additional file

Additional file 1: The clincal database and UGT1A/DPYD polymorphisms of all study patients. (XLSX 141 kb)

## Abbreviations

CI: Confidence internal
DCR: Disease control rate
IQR: Interquartile range
IRI: Irinotecan
mCRC: Metastatic colorectal cancer
NA: Non-acquired
OR: Odds ratio
ORR: Objective response rate
OS: Overall survival
PFS: Progression free survival
RESCIST: Response evaluation criteria in solid tumors

## References

[1] Garcia-Alfonso P, Chaves M, Muñoz A (2015). Capecitabine and irinotecan with bevacizumab 2-weekly for metastatic colorectal cancer: the phase II AVAXIRI study. BMC Cancer.

[2] Saltz LB, Cox JV, Blanke C (2000). Irinotecan plus fluorouracil and leucovorin for metastatic colorectal cancer. Irinotecan study group. N Engl J Med.

[3] Lamas MJ, Duran G, Balboa E (2012). The value of genetic polymorphisms to predict toxicity in metastatic colorectal patients with irinotecan⁃based regimens. Cancer Chemother Pharmacol.

[4] Miyata Y, Touyama T, Kusumi T (2016). UDP-glucuronosyltrans-ferase 1A1*6 and ∗28 polymorphisms as indicators of initial dose level of irinotecan to reduce risk of neutropenia in patients receiving FOLFIRI for colorectal cancer. Int J Clin Oncol.

[5] Gao J, Zhou J, Li Y (2013). UGT1A1∗6/∗28 polymorphisms could predict irinotecan induced severe neutropenia not diarrhea in Chinese colorectal cancer patients. Med Oncol.

[6] Cheng L, Li M, Hu J (2014). UGT1A1*6 polymorphisms are correlated with irinotecan-induced toxicity: a system review and meta-analysis in Asians. Cancer Chemother Pharmacol.

[7] Tsunedomi R, Hazama S, Fujita Y (2014). A novel system for predicting the toxicity of irinotecan based on statistical pattern recognition with UGT1A genotypes. Int J Oncol.

[8] Mego M, Chovanec J, Vochyanova-Andrezalova I (2015). Prevention of irinotecan induced diarrhea by probiotics: a randomized double blind, placebo controlled pilot study. Complement Ther Med.

[9] Yan L, Wang XF, Wei LM (2016). Effects of UGT1A1*6, UGT1A1*28, and ABCB1-3435C>T polymorphisms on irinotecan induced toxicity in Chinese cancer patients. Int J Clin Pharmacol Ther.

[10] Sunakawa Y, Ichikawa W, Fujita K (2011). UGT1A1*1/*28 and *1/*6 genotypes have no effects on the efficacy and toxicity of FOLFIRI in Japanese patients with advanced colorectal cancer. Cancer Chemother Pharmacol.

[11] Gentile G, Botticelli A, Lionetto L (2016). Genotype-phenotype correlations in 5-fluorouracil metabolism: a candidate DPYD haplotype to improve toxicity prediction. Pharmacogenomics J.

[12] Mazzuca F, Borro M, Botticelli A (2016). Pre-treatment evaluation of 5-fluorouracil degradation rate: association of poor and ultra-rapid metabolism with severe toxicity in a colorectal cancer patients cohort. Oncotarget.

[13] Etienne-Grimaldi MC, Boyer JC, Thomas F (2015). UGT1A1 genotype and irinotecan therapy: general review and implementation in routine practice. Fundam Clin Pharmacol.

[14] Leung HW, Chan AL (2015). Association and prediction of severe 5-fluorouracil toxicity with dihydropyrimidine dehydrogenase gene polymorphisms: a meta-analysis. Biomed Rep.

[15] Teh LK, Hamzah S, Hashim H (2013). Potential of dihydropyrimidine dehydrogenase genotypes in personalizing 5-fluorouracil therapy among colorectal cancer patients. Ther Drug Monit.

[16] Falvella FS, Cheli S, Martinetti A (2015). DPD and UGT1A1 deficiency in colorectal cancer patients receiving triplet chemotherapy with fluoropyrimidines, oxaliplatin and irinotecan. Br J Clin Pharmacol.

[17] Eisenhauer EA, Therasse P, Bogaerts J (2009). New response evaluation criteria in solid tumors: revised RECIST guideline (version 1.1). Eur J Cancer.

[18] Mik M, Berut M, Dziki L (2017). Right- and left-sided colon cancer – clinical and pathological differences of the disease entity in one organ. Arch Med Sci.

[19] Sakar B, Gumus M, Basaran M (2007). XELOX followed by XELIRI or the reverse sequence in advanced colorectal cancer. Oncology.

[20] Wang Y, Shen L, Xu N (2012). UGT1A1 predicts outcome in colorectal cancer treated with irinotecan and fluorouracil. World J Gastroenterol.

[21] Li M, Wang Z, Guo J (2014). Clinical significance of UGT1A1 gene polymorphisms on irinotecan-based regimens as the treatment in metastatic colorectal cancer. Onco Targets Ther.

[22] Xu C, Tang X, Qu Y (2016). UGT1A1, gene polymorphism is associated with toxicity and clinical efficacy of irinotecan-based chemotherapy in patients with advanced colorectal cancer. Cancer Chemother Pharmacol.

[23] Atasilp C, Chansriwong P, Sirachainan E (2016). Correlation of *UGT1A1*28* and **6* polymorphisms with irinotecan-induced neutropenia in Thai colorectal cancer patients. Drug Metab Pharmacokinet.

[24] Shimoyama S (2010). Pharmacogenetics of irinotecan: an ethnicity⁃based prediction of irinotecan adverse events. World J Gastrointest Surg.

[25] Fujita K, Ando Y, Nagashima F (2007). Genetic linkage of UGT1A7 and UGT1A9 polymorphisms to UGT1A1*6 is associated with reduced activity for SN-38 in Japanese patients with cancer. Cancer Chemother Pharmacol.

[26] Hazama S, Mishima H, Tsunedomi R (2013). UGT1A1∗6, 1A7 ∗3, and 1A9∗22 genotypes predict severe neutropenia in FOL⁃/FIRI⁃treated metastatic colorectal cancer in two prospective studies in Japan. Cancer Sci.

[27] Tziotou M, Kalotychou V, Ntokou A (2014). Polymorphisms of uridine glucuronosyltransferase gene and irinotecan toxicity: low dose does not protect from toxicity. Ecancermedicalscience.

[28] Carlini LE, Meropol NJ, Bever J (2005). *UGT1A7* and *UGT1A9* polymorphisms predict response and toxicity in colorectal cancer patients treated with capecitabine/irinotecan. Clin Cancer Res.

[29] Cecchin E, Innocenti F (2009). D Andrea M, et al. predictive role of the UGT1A1, UGT1A7, and UGT1A9 genetic variants and their haplotypes on the outcome of metastatic colorectal cancer patients treated with fluorouracil, leucovorin, and irinotecan. J Clin Oncol.

[30] Deenen MJ, Meulendijks D, Cats A (2016). Upfront genotyping of DPYD*2A to individualize Fluoropyrimidine therapy: a safety and cost analysis. J Clin Oncol.

[31] Sirachainan E, Reungwetwattana T, Wisetpanit Y (2012). Pharmacogenetic study of 5-FU-related severe toxicity in Thai cancer patients: a novel SNP detection. J Pharmacogenomics Pharmacoproteomics.

[32] Zhang XP, Bai ZB, Chen BA (2012). Polymorphisms of dihydropyrimidine dehydrogenase gene and clinical outcomes of gastric cancer patients treated with fluorouracil-based adjuvant chemotherapy in Chinese population. Chin Med J.

[33] Toffoli G, Cecchin E, Corona G (2006). The role of UGT1A1*28 polymorphism in the pharmacodynamics and pharmacokinetics of irinotecan in patients with metastatic colorectal cancer. J Clin Oncol.

[34] Lu CY, Huang CW, Wu IC (2015). Clinical implication of UGT1A1 promoter polymorphism for irinotecan dose escalation in metastatic colorectal cancer patients treated with bevacizumab combined with FOLFIRI in the first⁃line setting. Transl Oncol.

[35] Kobayashi M, Hazama S, Takahashi K (2012). Is there diversity among UGT1A1 polymorphism in Japan?. World J Gastrointest Oncol.

[36] Braun MS, Richman SD, Thompson L (2009). Association of Molecular Markers with Toxicity Outcomes in a randomized trial of chemotherapy for advanced colorectal cancer: the FOCUS trial. J Clin Oncol.

